# Optimal professional development ICT training initiatives at flagship universities

**DOI:** 10.1007/s10639-020-10154-y

**Published:** 2020-04-14

**Authors:** Xi Wang, W. James Jacob, Christopher C. Blakesley, Weiyan Xiong, Huiyuan Ye, Shangmou Xu, Fang Lu

**Affiliations:** 1 Department of Administrative and Policy Studies, School of Education, University of Pittsburgh, 156 Sterling Dr., Walnut, CA 91789 USA; 2 Collaborative Brain Trust, 45 W South Temple, Salt Lake City, UT 84101 USA; 3 Carnegie Mellon University, 135 Bucks Rd, Cranberry Twp, PA 16606 USA; 4 School of Graduate Studies, Lingnan University, AD208 Wong Administration Building, Lingnan University, 8 Castle Peak Road, Tuen Mun, Hong Kong; 5 Southern New Hampshire University, 2 Peabody Terrace #1804, Cambridge, MA 02138 USA; 6 Department of Administrative and Policy Studies, School of Education, University of Pittsburgh, 5500 Wesley W. Posvar Hall, 230 S Bouquet St, Pittsburgh, PA 15238 USA; 7 South China University of Technology, Room 1509, No.1 Building, No.381 Wushan Road, Tianhe Distric Guangzhou, 510641 People’s Republic of China

**Keywords:** Information and communication technology (ICT), Professional development, Human resources management, Leadership, World-class universities, Flagship universities

## Abstract

This study explores best practices and roles of information and communication technology (ICT) in select professional development centers at 16 flagship universities. Through adopting a qualitative case study design, this study explores the strengths and weaknesses of current technology training initiatives in the selected professional development centers. As part of the research and teaching programs at flagship universities, professional development center leaders shared about the current ICT practices as well as the strengths and limitations of their own centers. The analysis section includes a critical look at ICT practices among flagship universities from a human resource theory lens. Findings indicate common successes that facilitate the ICT practices of these centers including delivery mediums, services, ideas, and goals, as well as various barriers of implementing ICT training initiatives. The paper concludes with suggestions on how professional development center leaders, senior administrators, and educational policy makers can help improve professional development processes with the assistance of optimal ICT initiatives.

## Introduction

The rapid development in information and communication technologies (ICT) enhances teaching and learning experiences offered by higher education institutions (HEIs) (Stensaker et al. 2007). Because university sustainability depends upon the enrollment and retention of students, the effective integration of ICT plays a critical role in maintaining a competitive advantage. Previous research has discussed both the external and internal barriers that affect the process of technology integration, such as resources, training, and personal attitudes (Ertmer 1999; Nura et al. 2011). Research also advocates technology integration with pedagogical practices, rather than merely teaching certain technologies (Wang et al. 2014; Ambrose et al. 2010). While the examination of processes and outcomes of technology integration from faculty members’ perspectives is critical, Stensaker et al. (2007) highlighted the importance of top-level administrator support for organization-wide technology integration initiatives. Professional development organizations are often the best equipped to implement technology initiatives. However, there is a lack of empirical evidence as to whether current professional development centers (PDCs) are providing optimal technology training initiatives for their faculty and students. This study articulates the PDC leaders’ opinions about and their first-hand experiences in improving the technology integration process by addressing the following key research questions: First, how are information & communication technologies employed at flagship university professional development centers? Secondly, what are the barriers to implementing information and communication technologies at flagship universities?

This study explores the strengths and weaknesses of current technology training initiatives in selected flagship university professional development centers. Findings include examples on how to best advance technology initiatives in the professional development process. Suggestions are also provided to help readers understand how to overcome barriers that limit the integration of optimal ICT initiatives in different types of HEIs.

In order to help readers better understand the context of this study, we give the following definitions to some of the key terms used in this study. Wang et al. (2014) defined information and communication technologies (ICT) as “hardware and applications that help people to access, retrieve, process, and exchange information.” In the field of professional development, ICT can generally refer to faculty professional development related to computer-based devices Prestridge (2010a) and a “technologized” approach (Lankshear and Bigum 1998, p.12) to facilitating pedagogy (Loveless 2003). In this study, the concept of technology integration encompasses a variety of pedagogical methods and technologies related to computer use, including hardware, long-distance teaching and information platforms. For readability, this paper uses 1) the terms “ICT” and “technology” interchangeably, and 2) the terms “HEI” and “institution” interchangeably.

Many of this study’s participating institutions are generally positioned among the top 100 in the following global rankings: Shanghai Jiao Tong University Academic Ranking of World Universities (ARWU), *Times Higher Education* (*THE*), and Quacquarelli Symonds (QS) (Jacob et al. 2015). In this study, we use the term *flagship university* to address a more relevant model of leading national universities as advocated by Douglass (2016), Douglas and Hawkins (2016), and others (Yonezawa 2007; Gao 2015; Teferra 2016). The role of a flagship university is one that often meets world-class university standards as defined by the leading ranking systems. Flagship universities also include the top universities in a given economic zone, country, and or geographic region. In this regard, The University of the South Pacific is also a regional example as the leading university in 12 Pacific Island countries. While world-class universities are generally considered flagship universities, not all flagship universities meet the exclusive lists of top-ranked institutions.

## Literature review

### The transforming purpose for using ICT in education

It is widely recognized that the rapid change of technology has become a necessary component of enhancing the quality of education (Eynon 2005; Gaible and Burns 2005; Wang et al. 2014). Many HEIs are utilizing technology as one of the key initiatives in their professional development programs. However, current literature indicates various purposes for and ways of using technology by different institutions. Studies show that technology integration strategies differ across disciplines and curricular domains (Eynon 2005; Hsu et al. 2013). For example, Eynon (2005) found that the most common use of technology within higher education institutions was to provide students with a range of online resources. She also examined other academic motivations, such as enhancing the educational experience for students; compensating the changes happening in higher education, such as the increasing student numbers and demand for flexible learning opportunities; and personal interests and enjoyment. In contrast, other studies have found that the majority of teachers still use technology passively as a “learn-from” medium (Wang et al. 2014; Siefert et al. 2019). Bigum (2002) described this passive adoption of technology practices as “domesticating” the computer in classrooms. Prestridge (2010a) illustrated the practice as a “re-tooling” model that serves to strengthen teachers’ approaches to implementing technology, but without changing their pedagogy.

In addition to the use of technology for logistical efficiencies or to support passive learning practices, research also shows that there is a transformational rationale for using technology in education. As Becker (1994) noted, the best educational purpose for using technology should be to promote student-centered learning. After examining eight world-class universities’ faculty development centers, Jacob et al. (2015) concluded that instead of merely applying technology for its own sake, higher education institutions should implement technology to enhance teaching and learning endeavors. Further, Albion et al. (2015) argue that the integration of technology can *be* the educational innovation (as opposed to solely supporting or enhancing an existing endeavor). Some innovative pedagogical practices require teachers to implement technology to create an environment that supports learners’ cognitive processes in problem-solving. These cognitive processes can provide learners a variety of critical, creative, and complex thinking opportunities (Wang et al. 2014). Ertmer and Ottenbreit-Leftwich’s (2013) study suggested that the ultimate instructional and learning goal is to engage students in authentic problem-solving through the support of technology, rather than merely integrating technology into classrooms. Prestridge (2010b) concluded that ICT professional development should move from infrequent technology integration within a curriculum to a model that enables teachers to see the transforming potential of ICT, which is to integrate technology in promoting students’ substantive cognitive learning and problem-solving competencies.

### Role of research and innovation

Optimal technology initiatives can fundamentally facilitate innovative research among faculty members and students. Technology-based research initiatives are a core focus of PDC offerings at world class universities. Related research includes finding innovative and optimal approaches to teaching, designing curricula, and evaluating current practices. This evidence-based approach can improve teaching and learning with high relevance to faculty members’ and students’ actual needs (Jacob et al. 2015). The process of adopting evidence-based teaching and learning highlights what Albion et al. (2015) describe as the important “transformation” of research within higher education institutions. However, several factors, including teachers’ concepts of technology (Lim et al. 2013), policy and leadership issues (Wagner et al. 2005), can influence the transformation process.

Instructors’ traditional perceptions about teaching and learning with technology can hinder the process of transformation. The level at which teachers can accept technology in their teaching is described as “teachers’ digital literacy,” which is a complex concept (Albion et al. 2015). Digital literacy includes teachers’ broad views of the information era and consideration of constructing technology teaching models (Krumsvik 2008). The gap between research outcomes and technology usage in teaching is a result of the lack of transformation. This gap can also be described in terms of the relationship between research and practice or the complement between theoretical knowledge and experiential knowledge. For decades, there have been debates about the relationship between research and practice in education (Bereiter 2014). As a relatively new element of teaching, computer technology integration can be a driving force behind pedagogical innovation (Kozma and Vota 2014). Recent theoretical research and case studies suggest that the combination of research and practice is the optimal way to fill this gap between research outcomes and technology usage in teaching (Guri-Rosenblit 2002; Bereiter 2014). And although teachers are central to learning experiences, policymakers and center leaders should take responsibility for creating conditions that facilitate the transformation process and collaboration between faculties and technology researchers and experts (Kennisnet Foundation 2011).

### Barriers to implementing ICT in HEIs

A number of studies have examined different barriers that influence the implementing of technology in higher education institutions (Ertmer 1999; Eynon 2005; Hew and Brush 2007; Nura et al. 2011; Ertmer and Ottenbreit-Leftwich 2013; Romero Alonso et al. 2019). These studies have clarified a variety of barriers. This review draws upon the earliest one—Ertmer’s (1999) article, which divided the various barriers into two categories: external and internal. External barriers refer to the difficulties of implementing technology due to external reasons, such as resources, time, training, institutional support. In contrast, internal barriers occur when the obstacles to adopting technology come from teachers and students themselves (e.g., personal beliefs, attitudes, commitment, skills).

In reference to external barriers, Eynon (2005) argued that lack of time, less access to certain software, and problems with copyright due to prohibitive costs or permission time are three difficulties encountered by academics when using technologies for teaching and learning. He further noted that whether an institution has a strategic plan to implement ICT, whether the strategy meets the department’s and school’s specific needs, and whether schools have enough skilled IT staff are also major factors that may inhibit the implementation of ICTs in academics. Hew and Brush (2007) also concluded that a lack of resources—including limited hardware, time, and access—were the most commonly recognized barriers. Further, O'Meara and Terosky (2010) explained that faculty growth is driven both by the individuals’ needs and by specific socio-cultural, institutional, and personal contexts.

In addition to external barriers, internal barriers are also important and need to be addressed while implementing technology in teaching and learning contexts (Sang et al. 2010; Ertmer and Ottenbreit-Leftwich 2013; Romero Alonso et al. 2019). Nura et al. (2011) surveyed 312 higher education institution members of staff in Sokoto state, Nigeria, about their acceptance of the adoption of technology in human resource management. Results showed that when education professionals believe using technology will increase their effectiveness and job performance, they are more likely to put effort into learning about that technology. Palak and Walls (2009) found that even in those schools with rich technology, teachers’ strong traditional beliefs impeded the use of technology in teaching and learning. Therefore, examining teachers’ inner drive to implement technology and how higher education institutions help to stimulate teachers’ inner drive is crucial to implementing ICT in education. Little and Housand (2011) stated that support from school administration can ensure sufficient time and resources devoted to a professional development initiative. Albion et al. (2015) also pointed out, “in exploring the conditions for integration of ICT in education, there is a need for better understanding of the teachers’ role, and subsequently, a need to study professional development programs, models and strategies as a means to improve their impact on teachers’ practice” (p. 658).

Previous research discussed how to successfully implement technology in education. It also identified several barriers associated with the process. However, most of the current literature discusses the motivation and implementation of technology from teachers’ perspectives. For example, a common area of inquiry asks how teachers can best utilize technology in their classrooms to better engage students and achieve educational outcomes. Few of the reviewed research studies examined how a central professional development organization or programs can provide guidance in the process of implementing ICT. In order to better explore the conditions for the integration of ICT in education, there is a need for better understanding the professional development programs’ strategies and practices. Moreover, since technology integration is a topic that requires the most updated technological information, there is also a particular, continuing need for connecting research and current practices.

## Research design

We employed a case study approach to gain insights into current practices surrounding technology integration in faculty development centers. Luttrell (2010, p. 1) stated that qualitative research is “committed to participants using their own words to make sense of their lives; it places an importance on context and process.” We rely upon participants’ personal experiences to offer substantive interpretations (Luttrell 2010). Merriam (2009) pointed out that the qualitative case study shares characteristics of similar forms of qualitative research to search for meaning and understanding. She further stated that the qualitative case study could be characterized as being particularistic, descriptive, and heuristic (Merriam 2009, p. 43). This study focused on a specific phenomenon in a particular kind of organization. Specifically, technology integrated by PDCs is the phenomenon or, as Yin (2014) calls it, the “case” we were interested in investigating. Miles and Huberman (1994) indicate how case studies have “bounded context[s]” or “edge[s].” In this study, our “bounded context” or “edge” is the professional development center. We only examined how this particular type of central organization implemented technology. In addition, through deeply analyzing each participant’s response, this study gave a rich and thick description of the phenomenon under study. Further, by providing real experiences from the PDC leaders in regard to implementing technology, we believe this study will illuminate the readers’ understanding about how to effectively integrate technology into their own contexts.

### Participants

This study focused on current technology practices as well as strengths and weaknesses in world class universities. Therefore, purposeful sampling was conducted to recruit participants. The unit of analysis in this study is the professional development centers within the participating flagship universities. The majority of participating institutions were selected from the top 100 universities, on average, based upon the three most prominent global university ranking systems: Academic Ranking of World Universities (ARWU), *Times Higher Education* (*THE*), and Quacquarelli Symonds (QS). Multiple participating flagship universities were not ranked in the top 100 of these ranking systems (e.g. the University of the South Pacific and the University of Cape Town), yet all are highly regarded as prominent institutions in their respective regions, which meets our definition and selection criteria as flagship universities. These top-ranked HEIs were then organized by geographical region, such as Asia, Europe, Oceania, and the United States. Participants were further identified as those who are in leadership positions in faculty development centers in selected case universities. A total of 16 senior leaders were chosen to participate in this study. Most individuals interviewed had substantial experience in higher education professional development training based upon their public website profiles.

### Instrument

Data for the study was obtained from two sources: interviews and document analysis. Semi-structured interviews with open-ended questions were conducted with selected university faculty development leaders around the world. An interview questionnaire consisting of 13 questions was developed to serve as a guide for interviewing these leaders. An online questionnaire version of the instrument was also developed as an option for participants to complete if they chose to do so. Documents such as an institution’s organizational chart were chosen to provide a second data source and were used to cross-validate the interview questions.

### Data analysis

Data was recorded, transcribed, cleaned and analyzed by open coding based upon the responses to the research questions on leadership perceptions of technology in faculty development centers. Qualitative data was analyzed with the assistance of a computer software program, NVivo, to allow common themes to emerge. Transcriptions were independently coded in reference to different interview questions. Then authors compared and contrasted the open coding across each university. Broad themes emerged based upon each interview question. These broad themes were further analyzed into subthemes, which then were categorized in the findings section (Maxwell 2013). A unique code list was used to decode qualitative responses of participants from each university. Each university was given a unique number (from ICT01-ICT12). Each interview question was assigned a unique number (from 01 to 13). For instance, ICT01–06 indicates that this data came from the first university in the unique code list and the participant’s response to interview question 6. In addition, a brief description of the PDC in each selected university was given to illustrate the participating universities, including their PDCs’ names and the geographic region of their university (see Table 1).

**Table 1:** Participating Flagship Universities, Juxtaposed to the Three Leading Global Ranking Systems

Flagship University	PDC Name	Region	Ranking System		
			ARWU (2018–19)	*THE* (2019)	QS (2019)
Australian National University	Centre for Higher Education, Learning and Teaching (CHELT)	Oceania	76	49	24
Carnegie Mellon University	Eberly Center for Teaching Excellence & Educational Innovation	Americas	95	24	46
Cornell University	Center of Teaching Excellence	Americas	13	19	14
Imperial College London	Educational Development Unit	Europe	24	9	8
London School of Econ-omics and Political Science	Teaching and Learning Centre	Europe	151–200	26	38
National University of Singapore	Office of Resource Planning	Asia	67	23	11
Oxford University	Educational Development, Oxford Learning Institute	Europe	7	1	5
Seoul National University	The Center for Teaching and Learning (CTL)	Asia	101–150	63	36
Technical University of Munich	PROLEHRE	Europe	52	44	61
University of Cape Town	Centre for Innovation in Learning and Teaching	Africa	201–300	156	200
University of Hong Kong	Centre for the Enhancement of Teaching and Learning	Asia	101–150	36	25
University of Melbourne	Center for the Study of Higher Education (CSHE)	Oceania	41	32	39
University of Pennsylvania	Center for Teaching and Learning	Americas	17	12	19
University of Pittsburgh	Center for Instructional Development and Distance Education (CIDDE)	Americas	89	110	136
University of the South Pacific		Oceania			
University of Tokyo	Center for Research and Development of Higher Education	Asia	25	42	23

*Sources:* Created by the authors with data from Quacquarelli Symonds (QS) 2019, Shanghai Jiao Tong University Academic Ranking of World Universities (ARWU) 2019, and *Times Higher Education* (*THE*) 2019 for the years noted in the references.

## Results

The purpose of this study was to examine how technology is being used in professional development centers in selected world class universities, and what strengths and weaknesses are associated with the technology implementation process. Through analyzing the data, we identified two broad themes based upon our two research inquiries: “the usage of technology in professional development centers” and “barriers.” After further analysis of the participants’ responses, five subthemes emerged under these two broad themes, which were 1) purpose of technology training, 2) the pedagogical side of technology training, 3) various formats of technology training, 4) external barriers and 5) internal barriers. The following section presents these broad themes by subdividing them according to the five subthemes.

### The use of ICT in professional development centers

#### Purpose of ICT training

Across all these universities’ PDCs, the main purpose of using technology is to enhance teaching and learning. Therefore, technology is often seen as the thing that can help to transform teaching practices. Most of the participants responded that using technology in their centers focuses on how to fulfill good educational principles. They used different technologies, instead of merely providing instruction about how to use a specific technology. As the PDC Director from Carnegie Mellon University responded:

What we are always trying to do is find opportunities where technology can help solve a teaching or learning problem, or where technology can open a new opportunity to improve something that might not already be going on. So, we really look for technology not just for technology sake, but really…in order to improve teaching and learning. And we really focus on the learning side, and thinking about that very deliberately. (ICT02–09)

The Imperial College London Director also addressed how technology is used in his center:

We offer some support in terms of the pedagogy of using e-learning and learning technology, and tools and technologies of that type related to learning. We don’t train each staff or students in the mechanics of using functions provided by our IT Center, who do the training on technology. We just support the pedagogy. (ICT10–06)

This training purpose is also addressed by the University of Pennsylvania Executive Director, as he described:

The clear strength I think is that we are very good about thinking about the use of technology for the teaching end, and not getting caught up by thinking technology is an end to itself. I think that is a real positive. So we are able to help faculty and graduate students really think about what they are trying to accomplish in their teaching and then how to use technology to do that rather than start with what technology is out there and what you might use it for. (ICT07–06)

The basis for this transforming purpose of using technology is that many PDC leaders have realized that technology is just a learning tool. Thus, only teaching with new technologies is less meaningful to faculty members. Technology cannot be put before teaching and learning. Central PDCs should not only help faculty to integrate technologies; it is more important to inspire faculty members to think about ways to integrate technologies. To many PDC leaders, thinking about what learning objectives need to be achieved is more important than training faculty members in the use of new technologies. Therefore, technology is a tool that can enhance the teaching and learning experience. A prioritization of pedagogy serves the function of putting all these things together.

#### Pedagogical side of ICT training

Because the main purpose of using technology in these institutions is improve education and engage students, technology training typically addresses pedagogical issues. Usually the centers’ staff will offer consultations; listening to the faculty’s specific needs, and then advising on the incorporation of certain kinds of technologies to meet their students’ learning needs. Consultants ensure that faculty know how to apply technologies that are best connected with the educational conceptual perspectives. For example, as the Executive Director at the University of Pennsylvania explained: “We don’t tell people the ways or the technical aspects of how to use a particular technology, but we will lead a discussion on how they might use it, not the technical side, but the pedagogical side of it” (ICT07–06).

A more specific example given by the Director of the Center for Teaching Excellence at Cornell University in terms of how to provide technology support:

We are talking about teaching with technology, we are not talking about, like, how do I set up a blackboard account…what we are talking about is how to actually use that tool to perhaps help your students to succeed or overcome some of the challenges you might be facing in your teaching, for example, you are interested in making sure all the students are able to get the materials for class, perhaps, you may use blackboard… you might decide you want to have the opportunity to do the discussion outside the class, perhaps you will use piazza or the discussion boards that are in blackboard. You are teaching the large class, you want to have the opportunity to interact with your students, maybe use I-Clickers or Poll Everywhere, or Twitter. (ICT09–05)

This current trend is, further, in line with the previous literature on the transforming purpose of using ICT in education (Prestridge 2010a, b; Wang et al. 2014; Albion et al. 2015). Almost all the PDC leaders commented that their current use of technology is to integrate technology in creating a learning environment that enhances students’ critical thinking and cognitive learning.

#### Various formats of ICT training

Current technology training within the professional development faculty center happens in a variety of formats in terms of its organizational structure, training forms, and training content. Some centers have their own technical support group and provide centralized technology support to the whole university, while others may need to cooperate with a distinct group of people, who provide the mechanics of using technology. As the center leader in the Technical University of Munich responded:

We cooperated strongly with the media center that is called, at TUM it is the Center for Modern Media. They only look into the technological side of it. They don’t look into the didactics and pedagogy behind it; that is our job... Fortunately, we don’t need to worry about the technical side and all that support stuff. And we just offer the pedagogical knowledge behind it. (ICT14–06)

However, most of the center leaders responded that instructional technology is an integral part of the professional development activities provided by their centers. They support both faculty and students on how to use specific technologies and software in order to enhance teaching and learning.

In addition, the training forms are diverse. The two most common forms of training are workshops and seminars. However, many centers also provide a blend of ways to best help their faculty and students, such as individual consultation, TA training programs, house calls, or even directly going to faculty members’ offices or classrooms to offer technical support. For example, the Director from the University of Pittsburgh’s University Center for Teaching and Learning described how if faculty members do not know how to use a technology feature in their teaching, they reach out to the University of Pittsburgh’s PDC for help:

If faculty members don’t know how to use the grades center, they can call us. We will come to their office, and help. Usually we just help people on the phone because it’s fast, quicker than email.… We also offer workshops all year round.… We will conduct one-on-one meetings, or we will hold [training] classes on how to use [various] kinds of technologies. (ICT01–06)

The Associate Dean at the University of Melbourne’s Center for the Study of Higher Education also addressed their center’s flexible way in providing technology training to their faculty members:

We have a number of scholars here who are working on e-learning, and using online technologies, and researching with online technologies. Our particular strength is around research in this area. What we are trying to use more of is to have [more of] an online presence for some of our professional development activities, so that people can access them if they are unable to actually attend a professional development training session. (ICT08–07)

Moreover, these faculty PDCs also provide various types and content of technology training. Some of the most commonly used technologies and software include: smartphones, tablets, laptops, Learning Management Systems, Clickers, MOOCs, MOODLE, Blackboard, Canvas, and so forth. These various technology training platforms ensure that both faculty members and students can receive the best training services available to enhance teaching and learning. This finding indicates what previous literature recommended: that since there is no single technology option to meet different needs of both faculty members and students, institutions should not only offer an online support mode, but should blend delivery formats of technology support (Jacob et al. 2015).

### Barriers

Previous research clarified that different types of barriers exist, including internal barriers and external barriers (Ertmer 1999). However, the barriers for each center are quite different. The specific barrier type depends on their resources, policies, or traditions. In most cases, the leader of the center could successfully recognize barriers and find solutions. However, those identified barriers were usually complex issues with policy or historical underpinnings.

#### External barriers

A lack of resources is the main factor that influences the implementation of technology within faculty PDCs. As the University of Pittsburgh Director mentioned in her interview, a lack of resources restricted the development of technology in her center. Their best solution was to “balance against the broader needs of the university” (ICT01–07). Also, the Director of the University of Tokyo mentioned that the problem of budgeting “limited” (ICT12–07) the development of the ICT program in her center.

Although financial and physical resources are important in the development of technology integration, human capital resources such as staff, faculties and experts are a key influential factor. Staff who master the use of technology, faculties’ initiatives for technology, and researchers and experts who can bring more technical expertise to training are regarded as the three main aspects of implementing technology integration in faculty PDCs. Skilled staff members who have a broad knowledge of technology integration can facilitate better services for the faculty, as the Director of University of Pittsburgh mentioned in her interview.

We have people who have a very broad knowledge of educational technology. So when you have staff and a place like this, you need to have people who are inquisitive and who have a certain level of intellectual curiosity and want to know…. I think we have people who have solid skills that translate into better service toward faculty. (ICT01–07)

The central focus of technology initiatives is to help meet the needs of faculty members and students. As the Director of the University of Pennsylvania said about the faculties’ initiative for using technology, the responsibility of faculty PDCs is to assist faculty to apply technology according to requirements in their classes, instead of teaching them how to use technology.

The clear strength I think is that we are very good about thinking about the use of technology for the teaching end. And not getting caught up in thinking about technology as an end unto itself. I think that is a real positive. So we are able to help faculty and graduate students really think about what are they trying to accomplish in their teaching and then how to use technology to do that, rather than start with what technology is out there and what you might use it for. So that is the strength. (ICT07–07)

Also, as the Director of Imperial College London said about the faculties’ initiative, the use of technology can be individuation, given that the nature of the technologies is diverse.

The strength is the dispersed nature of the technologies, given that they are allowed to develop in a very organic way in the faculties. … Faculties are allowed to develop their own use of technology in e-learning as required and it is supplemented by centrally provided support and equipment and approaches. (ICT10–07)

Experts and researchers are the third aspect of the implementation of ICT. However, as the Director of the University of Pennsylvania said, cooperation between experts and researchers can be a big problem when a lack of expertise exists in their center.

But it also alludes to a weakness, which is that the technical expertise does not lie in our center, it lies elsewhere. So there are places where we might be more creative if we had greater technical expertise. But we don’t have it and that becomes a weakness. If I had my druthers, it would be great to hire somebody whose area of specialization was in instructional technology, who could really bring the technical expertise that we don’t have, but retain the pedagogical focus that we do have. (ICT07–07)

Under-skilled and underprepared human resources, as indicated by many directors of PDCs, is a main external barrier to implementation of ICT. Skilled staff, faculty members, and ICT experts who work closely together are among the most effective at the case study institutions. Faculty members from the teaching side generate the need for using technology and provide feedback about current needs to both staff and experts. Based on their feedback, staff could provide timely help or a long-term improvement workshop. Also, experts can use their knowledge and research to facilitate all the services from a PDC.

#### Internal barriers

PDC leaders at several universities, including in the UK, believe that they need little technology to facilitate their teaching delivery due to 1) the single location of their campuses, 2) the size of their classes, and 3) their traditional styles of teaching. The Director of Oxford University mentioned that the class size in their university is much smaller than other universities and face-to-face teaching is very convenient, and technology integration seems not to be so important:

I would say that because of the residential nature of our educational environment that technology has not been as important as it would be in other settings. We also have, with regard to students, we have very small class sizes compared to most other universities. So we simply don’t have as much need for a lot of intervening technology. You will walk down the hall to go to lunch and you might bump into your students, it is a very much more personalized face-to-face teaching environment. Even a lecture might only have 20 students. So we have not relied that heavily on technology ... and we don’t do a great deal of IT training in our office. (ICT05–08)

The Director of the London School of Economics and Political Science said that their constraints are physical space limitations relative to their number of computer experts.

I would say if I was looking at weaknesses I guess because of the nature of the disciplines we teach, there is no inherent drive to keep at the forefront of technology. We don’t have computer scientists in the institution …We have to introduce the latest … and encourage people to think about how they may or may not improve the quality of their interaction with students. I mean my other comment is that technology, for all its benefits, still fails to keep up with the ingenuity of human beings and providing their learning processes. So I would say technology still is not necessarily there, but we do what we can with it. (ICT06–07)

An Associate Professor from the Centre for Innovation in Learning and Teaching (CILT) at the University of Cape Town described this barrier as “insufficient visibility”. He said,

The university associates the department more strongly with its technological expertise (a marketing and communication set of issues), but shortage of staff in particular areas (e.g. monitoring and evaluation, learning design, curation and IP). (ICT19–07)

The Director at the Technical University of Munich pointed out another essential barrier in terms of the time conflict between research and teaching, which is also addressed by some other center leaders. He noted that people tend, especially when they are building their career, like in their thirty and forty. They tend to, you know, not focus too much on the teaching, just make sure the teaching goes long somehow. But where the merit comes from, that always the research side. And so, we, as a department of higher education, struggle a lot in making people giving their time to put effort into teaching. Because they say it doesn’t count, in the end, it doesn’t count. I won’t get merit for it. I would get merit for another publication but not for in that teaching side. (ICT14–07).

As recognized by previous research, internal barriers mainly represent the internal necessities of faculty members and students. However, our research found that the traditions and inherent drive of institutions, which determine the policy and tendency toward implementation of technology is an important aspect of internal barriers.

In most cases, diversified ICT training and services have become an important part of centers’ standard operating procedures, as they seek to enhance teaching and learning. Their primary motivation for using technology is to integrating technology with pedagogy in order to enhance students’ learning experiences. Related best practices have been described as using various tools to transform ways of teaching. However, the main external barriers that prevent the successful implementation of technology in PDCs are largely related to human resource deficiencies, including sufficiently skilled staff, motivated faculty, and experts in ICT (Stensaker et al. 2007). The internal barriers are much more needs-based and context specific.

## Discussion

This study explores the current use of technology in PDCs at selected flagship universities. Through examining how these PDCs use technology, we further identified both strengths and weaknesses of implementing technology across all the case study universities. Previous research emphasizes how to implement technology from teachers’ perspectives; we hope through investigating how technology is being adopted from a central organizational perspective, more institutional leaders and policymakers will draw some implications for supporting effective technology training at their institutions in an era of repeated disruptive innovations, processes, and challenges.

In all of these cases, it seems that providing technology training has already become an integral part of professional development activities in most of these PDCs. In addition, these centers address integrating technology with pedagogy to enhance students’ learning experiences. Further, this current trend is in line with previous literature on the transforming purpose of using technology in education (Wang et al. 2014; Albion et al. 2015; Byungura et al. 2016). As Prestridge (2010a, b) indicated, integrating technology should create a learning environment that enhances students’ critical thinking and cognitive learning. Our results show that this mission is one of the most important initiatives employed by those centers in flagship universities that provide faculty technology training. Therefore, if other universities also want to provide effective technology training to both faculty members and students, it is essential to address the pedagogical side of technology integration. Trainers may consider how a particular type of technology can best facilitate a specific teaching and learning need, rather than just teaching faculty members how to use that technology.

After investigating “why” these centers use technology, we further analyzed “how” they provide ICT training. In terms of how to implement ICT in education, Howie (2010) listed several activities to learn technology, including extended projects (weeks or longer), short task projects, self-accessed courses, teacher lectures and so forth. Little and Housand (2011) also provide several websites to show that online professional development activities provide increased learning opportunities for teachers. Through comparing different centers’ current activities and programs for implementing technology, we identified various formats of training that are being employed by the centers: workshops, seminars, individual consultation, online training, and even direct visits to faculty members’ classrooms or offices.

Research showed that one of the external barriers in implementing technology is a lack of time (Hew and Brush 2007). Our study also indicated that faculty members usually are too busy to be involved in different kinds of technology training. These diverse formats and approaches are in essence providing more opportunities for faculty members to utilize technology in their teaching. Previous research has discussed technology as a tool that helps with learning. For example, Biancarosa and Griffiths (2012) examined how hardware (e.g. smartphones and laptops) and software (e.g. applications and programs) foster students’ learning. However, the research seldom mentioned in detail the specific kinds of technologies that are widely used in current flagship universities - especially the software. We found that these prominent universities are primarily using Clickers, MOOCs, and Moodle across the world. Other higher education technology training centers may also consider using these globally-popular technologies to facilitate learning and engage students so that more learning resources can be accessed by and shared within different types of higher education institutions.

Internal barriers, including obstacles to teachers and students adopting technology, have been proven to be a key factor that influences the usage of technology (Sang et al. 2010). However, our research shows that faculty member and student needs for technology are not the only internal barrier to adoption. Many of the needs and motivations are often based on higher education stakeholders’ previous experiences (Martin et al. 2011) and traditions (Krumsvik 2008). Regardless of what resource restrictions exist, internal needs and motivations largely determine whether or not an institution develops optimal technology initiative. If the administrators decide not to apply technology integration in their institution based on their concerns about a tradition or real situation, their center may not pay much attention to technology.

Many studies have put forward different kinds of external barriers that influence the implementation technology in higher education institutions (Ertmer et al. 2012; Ertmer and Ottenbreit-Leftwich 2013). Our research shows that human resources have been regarded as the most influential external factor. Specifically, skilled staff, motivated faculty members, and experts and researchers in technology are three main human resources factors. The role of research has been highly valued by faculty development centers. There is no doubt that most directors of centers believe that research could facilitate implementation of ICT in teaching and learning. Previous research indicates that the best practice for transformation is to combine teaching and research (Guri-Rosenblit 2002; Bereiter 2014). Our research shows that the major problem in transformation in flagship universities is a lack of experts in faculty PDCs. Some directors indicated that there were technology experts outside their centers. However, the problem comes from the collaboration between centers and experts. Many previous studies have indicated that faculty members can benefit from sharing knowledge (Leask and Younie 2013; Twining et al. 2013). We believe that collaboration should not be confined to bilateral collaboration. Instead, the whole process of collaboration should focus on specific needs in research, teaching, and learning (Hermans et al. 2008; Prestridge 2010b). Also, as Tondeur et al. (2010) discussed, leaders and ICT coordinators can generally play a critical role. The directors of PDCs should take the lead in facilitating the implementation of ICT and collaboration among faculty members, centers, and experts.

## Conclusion and recommendations

Based upon the results, we conclude that professional development centers are key in providing optimal technology faculty-training initiatives at flagship universities. In exploring the conditions for integrating technology in these flagship universities, we developed four broad recommendations. We hope that these recommendations will be applied by different types of higher education institutions as regards the effective integration of ICT in higher education across different countries.

First and foremost, instructional technology has become a trend in higher education. This requires that centers provide technology training for the purpose of enhancing teaching and learning experiences, instead of only teaching how to use a specific type of technology.

Second, different approaches should be developed to support effective technology integration. Faculty members usually have specific needs regarding the utilizing of technology in their classes, so professional technology training should provide comprehensive consultation before supporting technology. It is essential to ask what the faculty members’ real needs are to provide the most effective technology support.

Third, multilateral collaboration among staff, faculty members, experts, and administrators guarantees the development of optimal ICT in professional development centers. Center leaders and administrators of HEIs should take responsibility to create conditions amenable to facilitating collaboration between experts and staff to provide better services for faculty members.

Fourth, administrators and faculty members should take a broad view of using technology; a view that is not restricted to any single delivery method, such as long-distance teaching. However, many optimal technology-based teaching and learning initiatives apply an integrated approach of pedagogy of delivery. Also, institutions with different cultures, traditions, and situations can find their optimum technology practices, which are context specific and based upon local needs.

This paper has illustrated how optimal technology initiatives are currently used in professional development centers among various flagship universities. We found that integrating technology with pedagogy to enhance students’ learning experiences is a prominent initiative being employed by most centers. Moreover, they are providing diverse formats of technology training, such as training a variety of participants, offering the latest instructional technologies, and conducting multiple training activities. These various forms of ICT training provide ample opportunities for faculty and students to enhance their teaching and learning experiences. The barriers to implementation of technology in such centers are diverse. The lack of available human capital resources—including skilled staff, motived faculty, and experts in ICT integration—and disruptive innovations and circumstances (e.g., the COVID-19 pandemic) are key external barriers. Also, internal barriers driven by the needs of teachers, students, or institutions are important and need to be addressed in implementing technology integration in teaching and learning (Sang et al. 2010).
